# Development of glaucoma predictive model and risk factors assessment based on supervised models

**DOI:** 10.1186/s13040-021-00281-8

**Published:** 2021-11-24

**Authors:** Mahyar Sharifi, Toktam Khatibi, Mohammad Hassan Emamian, Somayeh Sadat, Hassan Hashemi, Akbar Fotouhi

**Affiliations:** 1 School of Industrial and Systems Engineering, Tarbiat Modares University, Tehran, Iran; 2 Ophthalmic Epidemiology Research Center, Shahroud University of Medical Sciences, Shahroud, Iran; 3 Centre for Analytics and Artificial Intelligence Engineering, University of Toronto, Toronto, Canada; 4 Noor Ophthalmology Research Center, Noor Eye Hospital, Tehran, Iran; 5 Department of Epidemiology and Biostatistics, School of Public Health, Tehran University of Medical Sciences, Tehran, Iran

**Keywords:** Ophthalmology, Data Mining, Imbalanced Learning, Feature selection, Ensemble classification

## Abstract

**Objectives:**

To develop and to propose a machine learning model for predicting glaucoma and identifying its risk factors.

**Method:**

Data analysis pipeline is designed for this study based on Cross-Industry Standard Process for Data Mining (CRISP-DM) methodology. The main steps of the pipeline include data sampling, preprocessing, classification and evaluation and validation. Data sampling for providing the training dataset was performed with balanced sampling based on over-sampling and under-sampling methods. Data preprocessing steps were missing value imputation and normalization. For classification step, several machine learning models were designed for predicting glaucoma including Decision Trees (DTs), K-Nearest Neighbors (K-NN), Support Vector Machines (SVM), Random Forests (RFs), Extra Trees (ETs) and Bagging Ensemble methods. Moreover, in the classification step, a novel stacking ensemble model is designed and proposed using the superior classifiers.

**Results:**

The data were from Shahroud Eye Cohort Study including demographic and ophthalmology data for 5190 participants aged 40-64 living in Shahroud, northeast Iran. The main variables considered in this dataset were 67 demographics, ophthalmologic, optometric, perimetry, and biometry features for 4561 people, including 4474 non-glaucoma participants and 87 glaucoma patients. Experimental results show that DTs and RFs trained based on under-sampling of the training dataset have superior performance for predicting glaucoma than the compared single classifiers and bagging ensemble methods with the average accuracy of 87.61 and 88.87, the sensitivity of 73.80 and 72.35, specificity of 87.88 and 89.10 and area under the curve (AUC) of 91.04 and 94.53, respectively. The proposed stacking ensemble has an average accuracy of 83.56, a sensitivity of 82.21, a specificity of 81.32, and an AUC of 88.54.

**Conclusions:**

In this study, a machine learning model is proposed and developed to predict glaucoma disease among persons aged 40-64. Top predictors in this study considered features for discriminating and predicting non-glaucoma persons from glaucoma patients include the number of the visual field detect on perimetry, vertical cup to disk ratio, white to white diameter, systolic blood pressure, pupil barycenter on Y coordinate, age, and axial length.

## Introduction

Glaucoma is the second cause of irreversible blindness and the fourth cause of Moderate and Severe Vision Impairment (MSVI) in the world.[1] Glaucoma prevalence rises with age increasing, and it is one of the main risk factors for blindness and MSVI in people older than 50.[1].

The average number of persons who go blind from glaucoma has been increased from 2.5 million persons in 1990 to 3 million persons in 2015. Moreover, the average number of persons and the average number of persons who suffer from MSVI due to glaucoma has been raised from 3 million persons to 4 million persons from 1990 till 2015.[2].

It has been predicted that the number of persons suffering from glaucoma aged between 40 and 80 years old would be increased from 64.3 million persons in 2013 to 76 million persons in 2020 and 111.8 million persons in 2040.[3].

Previous studies have identified different risk factors such as age, gender, race, Interocular Pressure (IOP), diabetes, and family history for glaucoma.[4–7].

Among different studies performed in the population older than 40, the glaucoma prevalence has been reported from 1.44 to 4 %. In contrast, more than 50 % of patients suffering from glaucoma have been unaware of their illness.[5, 8, 9] The identified risk factors for glaucoma in Iran are age, IOP, diabetes, axial length, and gender.[5].

Glaucoma is usually an asymptomatic disorder. If a severe visual impairment is not detected, the patient will not be aware of their disease. Therefore, some physicians have called glaucoma the silent thief of sight.[10].

The blindness occurring due to glaucoma is irreversible, but some early treatment such as reducing IOP and some surgical interventions can cease blindness due to glaucoma. However, some early treatment activities such as reducing IOP and some surgical operations can help control the disease progression. Early detection and diagnosis of glaucoma and identifying high-risk groups can reduce the irrecoverable adverse effects of glaucoma.

Artificial intelligence and machine learning methods have various applications in solving medical and healthcare problems[11–13], such as in ophthalmology[14]. Developing automatic methods for ophthalmological diseases[15], ophthalmologic image analysis[16], network analysis for gene expression data for eye diseases[17], predicting the progressions of ophthalmologic diseases[18], and evaluating eye diseases’ progression[19–21] are some of these applications.

The automatic methods for predicting and diagnosing glaucoma can be used as the computer-assisted diagnosis (CAD) method and decision support system (DSS) to improve glaucoma diagnosis and management accuracy. In this study, the main aim is to design and develop machine learning models for glaucoma prediction. For this purpose, demographic characteristics, optometry, biometry, perimetry features, and ophthalmologic examination results are used as the input variables.

The main novelty of this study lies in several folds, including:

- Proposing and designing a two-step classification task: The first step includes training the base and single classifiers on the training dataset and evaluating their performance based on a subset of the training dataset named as validation dataset, finding the superior classifiers. The second step is designing a novel stacked ensemble classifiers based on the superior classifiers.
- Using comprehensive ophthalmology features like perimetry and biometry to develop the glaucoma prediction model without any fundus features. Features of this study come from non-interventional ophthalmologic examinations.
- To address a highly imbalanced dataset with 87 instances in the glaucoma class (1.9 % of all instances) without generating artificial instances.
- Analysing a cohort dataset.

### Literature review

Different methods based on artificial intelligence and machine learning have been used in various applications for glaucoma management in recent years. [22] For instance, building glaucoma interaction networks [17], assessing the optic disk [23, 24], and detecting visual field progression[19–21], diagnosis, and screening.[10, 11, 25–33].

To propose and develop models to screen and diagnose diseases using structured datasets and complex data such as medical images and gene expression data can assist the physicians in early managing and diagnosing glaucoma. [10, 11, 31, 32]

Table 1 summarizes the related works considering model development for screening and/or diagnosis of glaucoma.

**Table 1:** Summary of the previous studies considering glaucoma prediction, screening and/or diagnosis

Authors	Target		Type of Dataset	Dataset	Classifier
	Diagnosis	Do Screening or Not			
(Li et al., 2019)[31]	Yes	Yes	Structured	SAP Data	LDA, SVM, NB, ANN
(Liu et al., 2013)[10]	Yes	Yes	Structured, Images and Genes	Personal Data, Fundus Images, Genome Data	SVM MKL
(Li et al., 2018)[11]	Yes	Yes	Structured	Visual Field Repots	SVM, RF, K-NN, CNN
(Noronha et al., 2019)[25]	Yes	No	Images	Fundus Image	SVM, NB
(Yo and Hong, 2015)[28]	Yes	No	Structured	Clinical Variables	MLR, ANN
(Li et al., 2020)[29]	Yes	Yes	Structured and Images	Fundus Image, Medical History Data	RNN(ResNet101)
(Acharya et al., 2017)[26]	Yes	No	Images	Fundus Images	DT, QDA, LDA, SVM, KNN, PNN
(Mookiah et al., 2012)[27]	Yes	Yes	Images	Fundus Images	SVM
(Chai et al., 2018)[32]	Yes	No	Structured and Images	Fundus Images, Clinical Data	Multi-Branch Neural Network
(Pathan et al., 2021)[33]	Yes	Yes	Images	Fundus Images	ANN, SVM, AdaBoost
(Kim et al., 2017)[30]	Yes	No	Structured	RNFL Thickness, Visual Field test Parameter, General Ophthalmic Examination	RF, DT, SVM, KNN

SAP: Standard Automated Perimetry, LDA: Linear Discriminant Analysis, SVM: Support Vector Machine, NB: Naïve Bayes, ANN: Artificial Neural Networks, MKL: Multi Kernel Learning, RF: Random Forest, K-NN: K-Nearest Neighbor, CNN: Convolutional Neural Networks, MLR: Multi Logistic Regression, RNN: Residual Neural Network, QDA: Quadratic Linear Regression, PNN: Probabilistic Neural Networks, Reg: Regression, RNFL: Retinal Nerve Fiber Layer, IOP: Intraocular Pressure.

As listed in Table 1, several previous studies have proposed models for diagnosing and screening glaucoma using different features and datasets. The mentioned models can be used to assist physicians in early diagnosis and decision-making. Fundus images in most previous studies were used as the vital input data for developing glaucoma diagnosing and screening models. In a few previous studies, genome data were used to improve glaucoma diagnosing and screening models because genetic and race are two common risk factors for glaucoma identified in previous researches. Although fundus images and genome data have an excellent performance in diagnosing glaucoma, these data types increase the costs and complexity of models. These two data types with some structured data from other ophthalmologic examinations and clinical data can improve the performance of models.

This study used different features, including demographic characteristics, optometry, biometry, perimetry, and ophthalmologic examination results, to help glaucoma prediction.

## Materials and methods

**Fig. 1:**
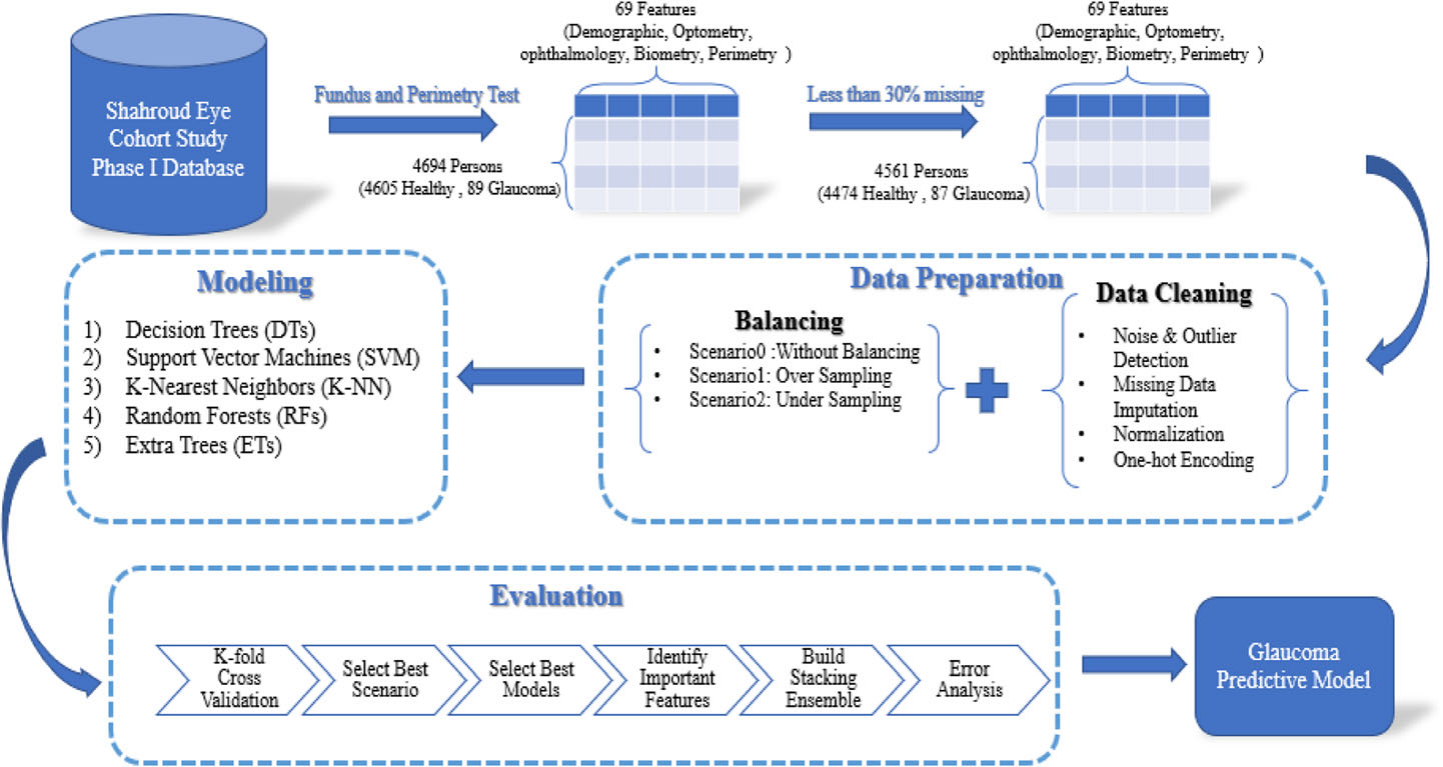
The main steps of this study proposed method for glaucoma prediction from Shahroud eye cohort study dataset

In this study, the ‘Shahroud eye cohort study’ dataset [34] is analyzed for predicting glaucoma based on Cross-Industry Standard Process for Data Mining (CRISP-DM) [35] methodology. Figure 1 shows the main steps of this study and the proposed method to predict glaucoma.

All preprocessing, modeling, and evaluation were done in Python language, and visualization was done in Python and Microsoft Excel in this study.

The steps as shown in Fig. 1 are described with more details in the following subsections:

### Data description

Shahroud eye cohort study was started in 2009 to diagnose and detect visual impairments and eye diseases in Shahroud city, Northeast Iran.[34].

In the first phase of the Shahroud eye cohort study, 6311 people aged 40-64 years were selected by random cluster sampling. Among them, 5190 individuals, including 2990 females, have been participated in the study. Fundus imaging and perimetry examination have been performed for 4694 participants. Assessing fundus and perimetry images by the ophthalmologists for glaucoma detection was performed. According to this study, the prevalence rate of glaucoma was estimated at 1.92 % of the population[5] among the considered persons, eighty-nine participants diagnosed as glaucoma patients.

Table 2 lists the demographic characteristics, optometry, ophthalmology, biometry, and perimetry examinations for the contributors in the first phase of the Shahroud eye cohort study.

**Table 2:** List of the considered variables in this study

Feature Name	Values	Feature Name	Values
Sex	Female/Male	Age (in Years)	Number
Body Mass Index (in kg/m2)	Number	Occupation Status	Nominal (including six categories)
Socioeconomic Status	Number	Marital Status	Nominal (including four categories)
Smoking	Yes/No	Diabetes	Yes/No
Diabetes Drug	Yes/No	Iris Color*	Nominal (including five categories)
Systolic Blood Pressure (in mm/Hg)	Number	Diastolic Blood Pressure (in mm/Hg)	Number
Hyper Tension Drug	Yes/No	Intraocular Pressure* (in mm/Hg)	Number
Visual Accuity* (in logMAR)	Number	Myopia*	Yes/No
Hyperopia	Yes/No	Nuclear Cataract*	Nominal (including five categories)
Glaucoma Drug*	Yes/No	Cortical Cataract*	Nominal (including five categories)
Glaucoma*	Yes/No	Posterior Subcapsular Cataract*	Nominal (including five categories)
Astigmatism* (in Diopter)	Number	Spherical Equivalent*(in Diopter)	Number
Angle Closure*	Yes/No	Number of Visual Detect*	Number
Vertical Cup to Disk Ratio*	Number	Axial length* (in mm)	Number
Anterior Chamber Depth* (mm)	Number	Corneal Thickness *(in mm)	Number
Lens Thickness* (in mm)	Number	Corneal White to White Diameter* (in mm)	Number
Corneal Radius, Flat* (in mm)	Number	Corneal Radius, Steep* (in mm)	Number
Keratometry* (K1)	Number	Keratometry* (K2)	Number
Iris Barycentric-X-Coordinate*	Number	Iris Barycentric-Y-Coordinate*	Number
Pupil Barycentric-X-Coordinate*	Number	Pupil Barycentric-Y-Coordinate*	Number
Pupil Distance*(in mm)	Number		

^*^Stars show that the feature has been measured in both eyes.

However, some participants who have more than 30 % missing values have been eliminated from this study. Therefore, 67 variables describing 4474 non-glaucoma persons and 87 glaucoma persons are considered in this study.

### Data preprocessing

The dataset should be partitioned into two non-overlapping datasets, including training and test datasets. For this purpose, K-fold Cross-Validation (C.V.) was used once with K=5 and K=10. The main steps of data preprocessing and preparation performed in this study were divided into two main categories: data cleaning and balanced sampling. Data cleaning steps are outlier detection and removal, missing value handling, data normalization, and one-hot encoding. Three different scenarios were evaluated and compared for data balancing or not. The first scenario was imbalanced data. The second and third scenarios used over-sampling and under-sampling strategies for balancing the training dataset.

For outlier detection, numerical variables are analyzed using the interquartile range (IQR) as a commonly used outlier detection method[36]. According to this method, no outlier is detected in numerical variables.

Categorical nominal variables were converted to dummy binary variables. Missing value imputation was performed for variables having a missing value rate lower than 30 %, and other variables were excluded from the study. Missing values were replaced with mean and mode for numerical variables and binary variables, respectively.

Features in our analysed dataset have different units or come from different examinations, and features have different value range. Some learning algorithms, similar distance-based methods like K-Nearest Neighbors (K-NN) or kernel machines like Support-Vector Machines (SVM), are sensitive to features ranges. This sensitivity can cause models bias to features with higher variations. Data normalization was applied to the Min-Max normalization method to avoid dominating variables with a low, extensive range of variations and improve evaluation metrics.

Since our analyzed dataset’s glaucoma prevalence rate was about 1.9 %, the class distribution was significantly imbalanced. On the other hand, more than 92 % of the considered persons belong to the non-glaucoma group (majority class), and only 1.9 % of the persons are assigned to the glaucoma class (minor class). The previous studies have shown that the classifiers trained on imbalanced datasets can have higher accuracy for classifying the major class. However, the minor class cannot be trained with high accuracy [37].

To overcome the imbalance distribution in this study, dataset between the classes, three different strategies applied were under-sampling, over-sampling, and combining over-sampling and under-sampling strategy used in the previous studies [37].

In this study, three different scenarios were designed and compared to address the imbalance dataset’s challenges. The first was sampling from data without balancing the class distribution (Scenario 0). The second was uses over-sampling from the minor class (Scenario1). The last was the balanced bagging ensemble method, which has been proposed to overcome the imbalance dataset challenges in a previous study (Scenario 2) [38]. The bagging ensemble using a high number of estimators can guarantee that the major class observations contribute to training one of the estimators.

### Modeling

Classifiers considered in this study include well-known classifier Decision Trees (DT) [39], Support Vector Machines, and ensemble classifiers having DT as their base classifiers, such as Extra Tree (ET) [40] and Random Forests (RF) [41]. These classifiers have different advantages and were used as a base classifier in three scenarios. For tuning the hyperparameters of the classifiers and choosing the resampling ratio, the Grid search method was used in this study. For DT and the ensemble based on DT like RF and ET, the splitting criterion was the ‘Information Gain,’ and the number of trees was 200. The kernel function of SVMs was ‘Radial Basis Function (RBF)’ in Scenario1 and Scenario2, and Polynomial’ in Senario3. The number of neighbours in the K-NN was seven, and the distance metric was ‘Euclidean.‘ In Scenario2, the oversampling ratio was different for each classifier and was determined by the Grid search. In Scenario3, the number of estimators of the balanced bagging ensemble method was 300. This number of the base estimator guarantee that every sample in the training set contributes at least to train one of the estimators and avoid overfitting.

### Evaluation

As illustrated in Fig. 1, evaluation tasks include the K-fold C.V. strategy for sampling from data, choosing the best scenario for handling imbalanced data, choosing the best classifiers, identifying the important features, building the proposed stacking ensemble model, and error analysis.

As mentioned in the preprocessing subsection, data was partitioned into training and test datasets based on the K-fold C.V. strategy for K=5 and K=10. The best scenario for handling imbalanced data and classifiers was chosen by comparing different combinations of the scenarios and classifiers based on their performance on the validation dataset. Then, important features ranked with the best classifiers were identified. Afterward, the proposed two-layered stacking ensemble classifier was built in which the first layer included different classifiers and the second layer used a logistic regression model.

For error analysis, evaluating and comparing the classifiers and different scenarios used for balanced sampling, various standard performance measures which were calculated and reported were accuracy sensitivity, specificity, F1-Score, and area under receiver operating characteristics (ROC) curve (AUC) as Eqs. (1)-(4).

1 $$Accuracy=\frac{TP+TN}{TP+TN+FP+FN}$$

2 $$Sensitivity= \frac{TP}{TP+FN}$$

3 $$Specificity= \frac{TN}{FP+TN}$$

4 $$F1-score= \frac{TP}{TP+ \frac{(FP+FN)}{2}}$$

### Experimental results

For evaluating the proposed scenarios, every classifier in each scenario was executed 30 times, and the performance measures, including accuracy, sensitivity, specificity, and F1-Score, are reported in Table 3.

**Table 3:** Comparing the performance of the classifiers in each scenario

Model	%Acc (Mean ± std)	%Spe(Mean ± std)	%Sen (Mean ± std)	%Sen (CI 95 %)	%F_score (Mean ± std)
	Scenario0_5fold				
DT	96.89 ± 0.45	98.35 ± 0.45	21.84 ± 9.32	(20.33, 23.35)	20.87 ± 8.20
SVM	98.08 ± 0.07	99.99 ± 0.04	00 ± 00	(00, 00)	00 ± 00
KNN	97.07 ± 0.34	98.91 ± 0.35	2.30 ± 3.46	(1.74, 2.86)	2.80 ± 4.23
RF	98.09 ± 0.05	100 ± 00	0.04 ± 0.48	(00, 0.12)	0.07 ± 0.90
ET	98.08 ± 0.07	99.99 ± 0.04	0.19 ± 1.04	(00, 00)	0.36 ± 1.95
Scenario0_10fold					
DT	96.90 ± 00.71	98.31 ± 00.68	24.86 ± 13.89	(23.28, 26.44)	23.24 ± 12.41
SVM	98.06 ± 00.00	99.98 ± 00.06	00 ± 00	(00, 00)	00 ± 00
KNN	97.04 ± 00.53	98.82 ± 0.53	02.25 ± 04.83	(1.70, 2.80)	2.66 ± 5.76
RF	98.09 ± 0.10	100 ± 00	0.04 ± 0.64	(0.00, 0.11)	0.07 ± 1.15
ET	98.08 ± 0.13	99.98 ± 0.06	0.27 ± 1.74	(0.07, 0.47)	0.48 ± 3.12
Scenario1_5fold					
DT	96.89 ± 0.48	98.35 ± 00	21.57 ± 9.53	(20.02, 23.11)	20.74 ± 8.28
SVM	97.61 ± 0.31	99.16 ± 0.33	17.94 ± 8.33	(16.59, 19.29)	21.75 ± 8.72
KNN	94.60 ± 0.61	96.23 ± 0.61	10.97 ± 7.23	(9.80, 12.14)	7.15 ± 4.72
RF	98.08 ± 0.06	99.99 ± 0.04	00 ± 00	(00, 00)	00 ± 00
ET	98.09 ± 0.06	100 ± 0.00	00 ± 00	(00, 00)	00 ± 00
Scenario1_10fold					
DT	96.81 ± 0.67	98.27 ± 0.64	21.82 ± 13.3	(20.31, 23.33)	20.39 ± 11.99
SVM	97.65 ± 0.47	99.21 ± 0.43	17.61 ± 12.1	(16.23, 18.99)	21.48 ± 13.61
KNN	94.60 ± 0.90	96.23 ± 0.91	10.99 ± 10.07	(9.84, 12.14)	7.13 ± 6.48
RF	98.08 ± 0.12	99.99 ± 0.05	0.08 ± 0.96	(0.00, 0.19)	0.14 ± 1.72
ET	98.09 ± 0.10	100 ± 00	00 ± 00	(00, 00)	00 ± 00
Scenari2_5fold					
DT	87.40 ± 1.49	87.65 ± 1.58	74.41 ± 9.25	(72.91, 75.91)	18.47 ± 2.27
SVM	83.52 ± 1.75	83.38 ± 1.79	65.35 ± 9.86	(63.75, 66.94)	13.22 ± 2.16
KNN	80.94 ± 1.59	81.80 ± 1.63	36.26 ± 10.05	(34.64, 37.89)	6.77 ± 1.81
RF	88.90 ± 1.08	89.24 ± 1.13	71.39 ± 8.96	(69.93, 72.84)	19.75 ± 2.42
ET	92.75 ± 0.91	93.49 ± 0.91	54.66 ± 10.67	(52.94, 56.39)	22.43 ± 4.41
Scenario2_10fold					
DT	87.61 ± 1.67	87.88 ± 1.72	73.80 ± 14.38	(72.16, 75.44)	18.61 ± 3.70
SVM	83.69 ± 1.80	84.02 ± 1.83	66.71 ± 15.36	(64.96, 68.46)	13.55 ± 3.16
KNN	81.00 ± 1.87	81.84 ± 1.91	37.69 ± 15.55	(35.90, 39.44)	7.02 ± 2.88
RF	88.87 ±1.48	89.10 ± 1.51	72.35 ± 13.67	(70.80, 73.91)	19.87 ± 4.00
ET	92.68 ± 1.16	93.40 ± 1.15	55.55 ± 16.60	(53.66, 57.44)	22.54 ± 6.68

Acc: Accuracy; Spe: Specificity; Sen: Sensitivity; CI: Confidence Interval

As illustrated by Table 3, the last scenario’s classifiers had better performance for predicting the non-glaucoma class and glaucoma class. Decision trees and random forests which outperform the other classifiers were contributed to the balanced bagging ensemble as its base classifiers.

**Fig. 2:**
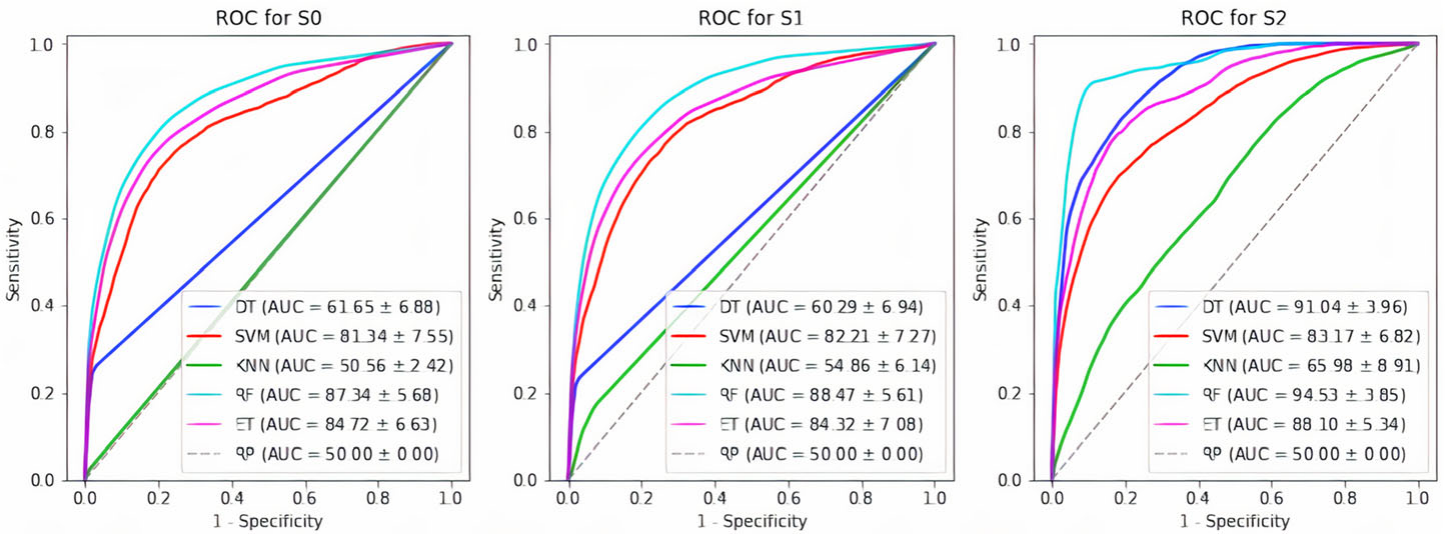
ROC curve for models in each scenario

The ROC curves are illustrated in Fig. 2 to compare different scenarios and classifiers.

According to DT and RF’s reasonable accuracy in the last scenario, top-ranked features were selected based on the feature importance scores assigned to the variables with DT and RF. 10-top ranked variables in the first quartile for glaucoma prediction from the Shahroud eye cohort dataset based on their average score on 300 executions of the classifiers are listed in Table 4.

**Table 4:** Feature importance in the best models

Rank	Decision Tree	Random Forest
1	NVD	NVD
2	VCDR	VCDR
3	Age	AL
4	AL	Systolic BP
5	WTW	Age
6	Systolic BP	AST
7	PCY	BMI
8	Spherical Equivalent	Diastolic BP
9	LT	PCY
10	AST	WTW

NVD: Number of Visual Detect; VCDR: Vertical Cup to Disk Ratio; AL: Axial Length; WTW: Corneal Whit to White Diameter; PCY: Posterior Capsule; LT: Lens Thickness; AST: Astigmatism, BMI: Body Mass Index; BP: Blood Pressure

As illustrated by Table 4, eight variables among 10-top ranked features identified by DT and RF are common, and they include NVD, VCDR, WTW, Systolic BP, PCY, AST, Age, and AL, which come from the different examination sources.

Finally, a novel two-layered stacking ensemble classifier is proposed in which the first layer combines two superior classifiers of the last scenario and the second layer uses logistic regression. Table 5 shows the performance measures of the proposed stacking ensemble classifier for Glaucoma prediction.

**Table 5:** Performance of stacking models

	%Acc (Mean ± std)	%Spe (Mean ± std)	%Sen (Mean ± std)	%Sen (CI 95 %)	%F_score (Mean ± std)
Stacking Ensemble trained with all features	83.56 ± 1.35	82.21 ± 1.75	81.32 ± 10.39	(80.07, 83.26)	80.98 ± 6.65
Stacking ensemble trained with top-ranked features	83.01± 1.98	83.00± 2.10	83.17± 12.03	(81.80,84.54)	82.58 ± 6.34

## Discussion

**Fig. 3:**
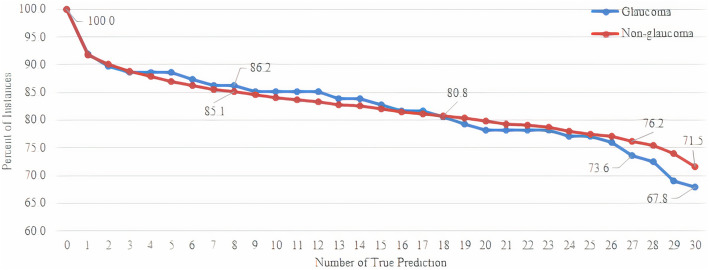
Number of true predictions for the data instances in 30 executions of 10-fold C.V. for the proposed stacking ensemble

For assessing the performance of the proposed stacking ensemble classifier in this study, 30 executions of 10-fold C.V. are analyzed. According to the experimental results, 3200 persons belonging to the non-glaucoma class (71.5 %) and 59 glaucoma persons (67.8 %) are correctly predicted in all executions. On the other hand, 368 persons of non-glaucoma class (8.2 %) and glaucoma class (8 %) are misclassified in all executions. Figure 3 indicates how many times of the executions each instance is predicted correctly.

According to Fig. 3, a novel confusion matrix (NCM) is proposed based on the thresholds for at least 0 %, 30 %, 60 %, 90 %, and 100 % instances that could be predicted correctly, as shown in Table 6. For example, if the threshold is 60 %, TP and TN show that the instances are correctly classified into positive (Glaucoma) and negative (non-glaucoma) at least 18 times of 30 executions, respectively.

**Table 6:** The novel proposed confusion matrix (NCM) for our proposed stacking ensemble method in this study

Threshold	TP	TN	FN	FP	%Acc	%Sen	%Spe
0	80	4106	7	368	91.78	91.95	91.77
30	74	3784	13	690	84.59	85.06	84.58
60	70	3614	17	860	80.77	80.46	80.78
90	64	3409	23	1065	76.15	73.56	76.20
100	59	3200	28	1274	71.45	67.82	71.52

The results shown in Table 5 are similar to the results corresponding to the threshold of 60 % in Table 6. According to the experimental results described in this section, data instances that no classifier can correctly predict negatively impact the classifier performance.

FP instances are the data instances with glaucoma, but the classifier has misclassified them as the non-glaucoma class label.

The average of top-ranked features for non-glaucoma and glaucoma classes is compared using the t-student test shown in Table 7 to investigate the significant difference between the non-glaucoma and glaucoma classes per each top-ranked feature.

**Table 7:** Comparing the average of top-ranked features for Non-glaucoma and Glaucoma classes using t-student test

Feature	Glaucoma	Non-glaucoma	P-value
NUMBEROFVISUALDEFECTOS	17.06	3.34	< 0.001
NUMBEROFVISUALDEFECTOD	18.75	2.48	< 0.001
Vertical CD Ratio Right	0.28	0.17	< 0.001
Vertical CD Ratio Left	0.28	0.17	< 0.001
Age	53.33	50.08	< 0.001
AL Left	23.51	23.04	< 0.001
AL Right	23.56	23.06	< 0.001
Systolic BP	130.77	128.02	< 0.001
Diastolic BP	79.79	79.44	0.403
AST Left	0.95	0.82	< 0.001
AST Right	0.98	0.82	< 0.001
BMI	28.3	28.45	0.415
PCY Left	-0.04	-0.02	0.007
PCY Right	-0.01	0.01	0.002
WTW Right	11.8	11.78	0.258
WTW Left	11.79	11.8	0.502
Spherical Equivalent	-0.69	0.1	< 0.001
LT Left	4.29	4.24	< 0.001
LT Right	4.31	4.26	< 0.001

AL: Axial Length, BP: BP: Blood Pressure, AST: Astigmatism, BMI: Body Mass Index, WTW: White to White distance, LT: Lenz Thickness

As listed in Table 7, the average of LT, Spherical Equivalent, AST, Systolic BP, AL, Age, VCD, and NVD are significantly different for glaucoma and non-glaucoma groups. It indicates that the mentioned variables can distinguish well two classes in our study. However, the average of WTW, PCY, BMI, and Diastolic BP is not significantly different for the glaucoma and non-glaucoma groups.

## Conclusions

Early identification of the persons with a high risk of glaucoma can help early beginning the necessary treatment and monitoring disease and prevent converting disease to the acute form. In this study, a novel stacking ensemble classifier composed of several machine learning classifiers is proposed, designed, and used for glaucoma prediction considering the Shahroud eye cohort dataset. This study’s input variables and predictors for glaucoma prediction are demographic characteristics, ophthalmology features, biometry, and perimetry descriptors for persons aged between 40 and 64 years old in Shahroud. Three scenarios are compared for handling an imbalanced dataset. The experimental results show that balanced bagging based on random forests and decision trees can improve the sensitivity and performance of glaucoma prediction with the average accuracy of 87.61 and 88.87, the sensitivity of 73.80 and 72.35, specificity of 87.88 and 89.10, and AUC of 91.04 and 94.53, respectively. On the other hand, the proposed stacking ensemble classifier achieves an average accuracy of 83.56, a sensitivity of 82.21, a specificity of 81.32, and an AUC of 88.54.

The previous studies used three different data types: fundus images, genome data, and structured data to develop a glaucoma prediction and diagnosis model. These studies achieved high-performance measures on fundus images or the combination of different data types, as shown in Table 8. This study used extensive ophthalmologic examinations like biometry, perimetry, and some clinical data to develop the predictive glaucoma model without fundus images or genome data. The developed model in this study has lower performance measures against other studies. Still, it has less complexity and cost and can use as the base of a decision support system in clinics to diagnose and screen glaucoma.

**Table 8:** Comparing the performance of the proposed method in this study with the previous studies

Authors	Dataset	Classifier	Best Performance Measures (%)
(Li et al., 2019)[31]	SAP Data	LDA, SVM, NB, ANN	AUC = 91.2
(Liu et al., 2013)[10]	Personal Data, Fundus Images, Genome Data	SVM MKL	AUC = 86.6
(Li et al., 2018)[11]	Visual Field Repots	SVM, RF, K-NN, CNN	Acc = 87.6, Sen = 93.2, Spe = 82.6
(Noronha et al., 2019)[25]	Fundus Image	SVM, NB	Acc = 92.65, Sen = 100, Spe = 92.0
(Yo and Hong, 2015)[28]	Clinical Variables	MLR, ANN	Acc = 84.0, Sen = 78.3, Spe = 85.9
(Li et al., 2020)[29]	Fundus Image, Medical History Data	RNN(ResNet101)	Acc = 96.5, Sen = 99.8, Spe = 99.9
(Kim et al., 2017)[30]	RNFL Thickness, VF test Parameter, General Ophthalmic Examination	RF, DT, SVM, KNN	Acc = 98, Sen =98.3, Spe = 97.5
(Acharya et al., 2017)[26]	Fundus Images	DT, QDA, LDA, SVM, KNN, PNN	Acc = 95.8
(Mookiah et al., 2012)[27]	Fundus Images	SVM	Acc = 95.0, Sen =93.33, Spe = 96.67
(Chai et al., 2018)[32]	Fundus Images, Clinical Data	Multi Branch Neural Network	Acc = 99.24, Sen = 97.91, Spe = 93.59
(Pathan et al., 2021)[33]	Fundus Images	ANN, SVM, AdaBoost	Acc = 98.0, Sen = 100, Spe = 97.0
This study	Extensive ophthalmologic examination and clinical data	DT, RF, ET, KNN, SVM, Stacking Ensemble	Acc = 83.56, Sen = 82.21, Spe = 81.32

SAP: Standard Automated Perimetry, LDA: Linear Discriminant Analysis, SVM: Support Vector Machine, NB: Naïve Bayes, ANN: Artificial Neural Networks, MKL: Multi Kernel Learning, RF: Random Forest, K-NN: K-Nearest Neighbor, CNN: Convolutional Neural Networks, MLR: Multi Logistic Regression, RNN: Residual Neural Network, QDA: Quadratic Linear Regression, PNN: Probabilistic Neural Networks, Reg: Regression, RNFL: Retinal Nerve Fiber Layer, IOP: Intraocular Pressure.

**Fig. 4:**
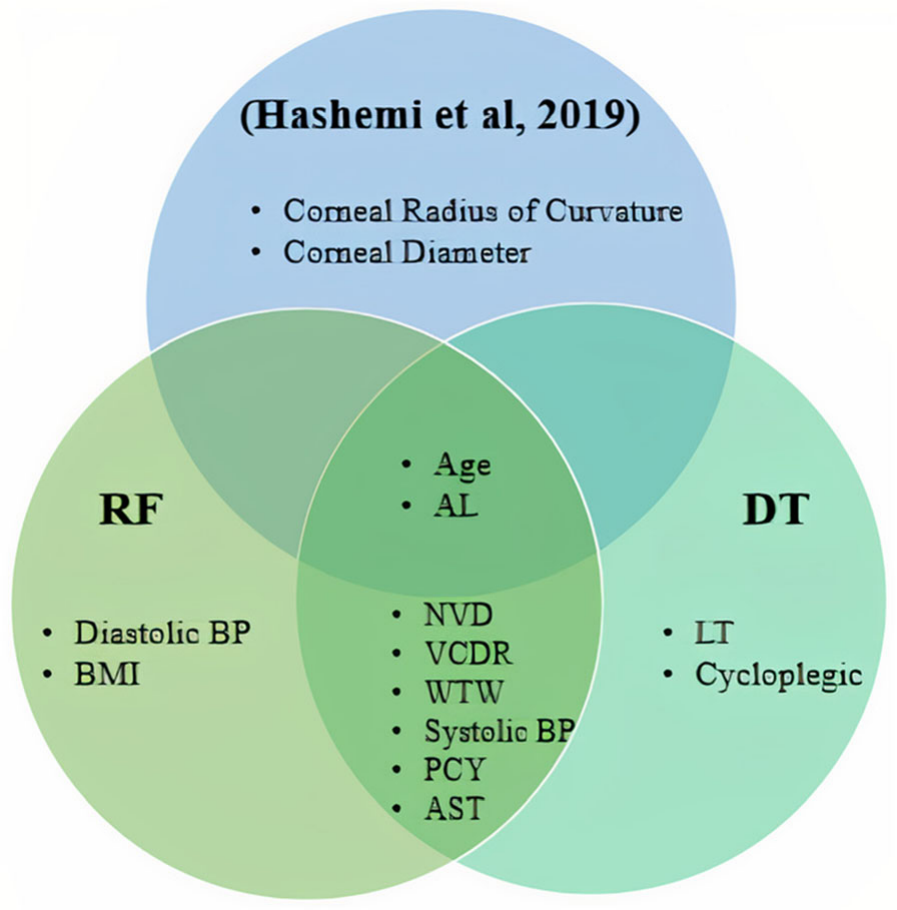
The Van diagram indicating our top-ranked features identified using DT and RF and top-ranked features identified in a previous study

Top-ranked features for predicting glaucoma identified using DT and RF are listed in Table 4. These top features come from different eye examinations like perimetry and biometry or demographic features that can measure in every clinic. As discussed in the introduction, some of these top features are the main risk factors of glaucoma diseases like age, BMI, blood pressure, and axial length, identified in many studies. On the other hand, some of these features like NVD and VCDR are used to identify glaucoma by physicians instantly. As mentioned in a previous study[5], top-ranked features for glaucoma prediction determined using simple and multivariate logistic regression have been age, IOP, sex, diabetes, myopia, and axial length. The Van diagram in Fig. 4 shows the top predictors of glaucoma.

This study aims to discriminate between glaucoma patients and non-glaucoma persons. The proposed and designed models in this study are disable to diagnose glaucoma type. Different types of glaucoma can be discriminated against, predicted, and diagnosed using machine learning models as a future research direction. Top-ranked features and risk factors for each type of glaucoma can be identified.

The first phase of the Shahroud Eye Cohort Study was used to predict glaucoma with extensive ophthalmologic examinations and demographic data without any fundus images and achieve an average accuracy of 83.56. The Shahroud Eye Cohort Study was conducted in two more phases with an interval of five years. For future work, the glaucoma condition of participating in the second phase can be use as the label for the first phase to develop a prognosis model which can identify people with glaucoma five years earlier and evaluate the model on the third phase.

This study has some main differences compared to the previous related works. In this study, different ophthalmological features are used as the input variables of our models, such as optometric examination results, biometric and perimetric features, ophthalmologic examinations. Moreover, top-ranked features include the variables describing ophthalmologic examination results. Using longitudinal data collected for 5-years provide us to assess the future trends and changes for glaucoma in people contributing to the cohort study.

## Data Availability

This is a retrospective study. Our considered dataset is a cohort dataset (Shahroud Eye Cohort Study). For. access to this dataset, legal procedure should be taken and written and signed commitment form should be. filled.

## Abbreviations

Acc: Accuracy
AUC: Area Under the Curve
ANN: Artificial Neural Networks
AST: Astigmatism
AL: Axial Length
BP: Blood Pressure
BMI: Body Mass Index
CAD: Computer-Assisted Diagnosis
CI: Confidence Interval
CNN: Convolutional Neural Networks
WTW: Whit to White Diameter
CRISP-DM: Cross-Industry Standard Process for Data Mining
C.V: Cross-Validation
DSS: Decision Support System
DT: Decision Tree
ET: Extra Trees
FN: False Negative
FP: False Positive
IOP: Interocular Pressure
IQR: Interquartile Range
K-NN: K-Nearest Neighbors
LT: Lens Thickness
LDA: Linear Discriminant Analysis
MSVI: Moderate and Severe Vision Impairment
MKL: Multi Kernel Learning
MLR: Multi Logistic Regression
NB: Naïve Bayes
NCM: Novel Confusion Matrix
NVD: Number of Visual Detect
PCY: Posterior Capsule
PNN: Probabilistic Neural Networks
QDA: Quadratic Linear Regression
RBF: Radial Basis Function
RF: Random Forest
ROC: Receiver Operating Characteristics
Reg: Regression
RNN: Residual Neural Network
RNFL: Retinal Nerve Fiber Layer
Sen: Sensitivity
Spe: Specificity
SAP: Standard Automated Perimetry
SVM: Support Vector Machines
TN: True Negative
TP: True Positive
VCDR: Vertical Cup to Disk Ratio
